# Morphological and p40 immunohistochemical analysis of squamous differentiation in endoscopic ultrasound guided fine needle biopsies of pancreatic ductal adenocarcinoma

**DOI:** 10.1038/s41598-021-00652-5

**Published:** 2021-10-28

**Authors:** Beate Haugk, David Horton, Kofi Oppong, John Leeds, Antony Darne, Philip Sloan, Thomas Ness, Claire Jones, Paul Bassett, Manu Nayar

**Affiliations:** 1 Department of Cellular Pathology, Royal Victoria Infirmary, Newcastle Upon Tyne Hospitals NHS Foundation Trust, Queen Victoria Road, Newcastle upon Tyne, NE1 4LP UK; 2 HPB Unit, Freeman Hospital, Newcastle Upon Tyne Hospitals NHS Foundation Trust, Newcastle upon Tyne, UK; 3 Statsconsultancy Ltd, Amersham, Bucks UK

**Keywords:** Cancer, Biomarkers, Medical research, Oncology

## Abstract

The basal-like molecular subtype of pancreatic ductal adenocarcinoma (PDAC) is associated with poor prognosis and upregulation in TP63ΔN (p40) network. Adenosquamous histology can be observed. This study assessed immunohistochemical p40 expression in fine needle biopsy (FNB) samples with PDAC and association with cytomorphological features of squamous differentiation and clinical data. 106 EUS FNBs with PDAC were assessed for eight cytomorphological features of squamous differentiation. P40 H-score (intensity 0–3 × percentage positive nuclei) was analysed for association with morphological features, patient age, gender, operability, chemotherapy and survival. P40 H-score in 14 paired FNBs and resections was compared. P40 h-score was 1–3 in 31%, 4–30 in 16% and > 30 in 13% of FNBs. It was significantly associated with intercellular bridges, elongated cell shape, sharp cell borders, angular nuclei with homogenous chromatin (p < 0.001) and dense cytoplasm (p = 0.002). Keratinisation was not seen. Inoperable patients (n = 81) had a shorter median survival for h-score > 30 (n = 9, 1.8 months) than for h-score ≤ 30 (n = 66, 6.7 months) not quite reaching statistical significance (p = 0.08). P40 was significantly associated with squamous morphology in FNBs with PDAC. P40 H-score > 30 showed a trend towards shorter survival in inoperable patients. Squamous differentiation may be a treatment target in PDAC.

## Introduction

Pancreatic cancer, often synonymously used with pancreatic ductal adenocarcinoma (PDAC), is one of the most lethal cancer types with an overall five-year survival rate of approximately 6%^1^. The incidence is rising^2^, and it is predicted to become the second leading cancer killer in the next decade^3^. Surgery remains the only potentially curative treatment for PDAC, but the 20% of patients operable at presentation will have an 80% recurrence rate^4^. Available systemic treatment options also have a limited overall impact^5^.

Over the past decade extensive work based mainly on resected PDAC tissues was performed to identify molecular subtypes of PDAC to facilitate precision medicine in order to improve outcome^6–9^. This has highlighted a complex molecular landscape in PDAC involving multiple genetic, epigenetic and tumour microenvironmental factors^5,9^ with significant heterogeneity between and within tumours and limited clinical value. Two main epithelial subtypes of PDAC appear to be emerging: (1) the basal-like/quasi-mesenchymal/squamous type which is mainly referred to as basal-like; this is more commonly associated with p53 mutation, upregulation in TP63ΔN (p40) network, and clinically poor prognosis. (2) the classical/progenitor type, referred to as classical, in which Guanine Nucleotide binding protein, Alpha Stimulating activity polypeptide (GNAS) mutations are more common and the outcome appears to be more favourable^10^. Molecular analyses have also identified different, prognostically relevant stroma types, including a partial association of basal-like phenotype with extra-cellular matrix-rich stroma indicating epithelial-stromal interaction^7,11,12^. Bailey et al. demonstrated an association of the squamous molecular type with adenosquamous histological subtype of PDAC which has long been recognised as a specific morphological subtype associated with worse outcome^8,13^.

The clinical diagnosis of PDAC changed significantly with the introduction of endoscopic ultrasound (EUS) guided fine needle aspiration (FNA) which has become the gold standard for the assessment of solid pancreatic lesions^14^. Technical developments over the last decade have resulted in new generation biopsy needles which provide fine needle biopsies (FNBs) with intact tissue fragments showing higher accuracy in the diagnosis of PDAC and providing paraffin embedded intact tissue for ancillary tests^15^.

DeltaNp63 (p40) is one of 10 p63 isoforms that has been shown to be a reliable and specific marker of squamous differentiation in pancreatic tissues^16^. The aim of the present study was to evaluate consecutive PDAC FNB samples for cytomorphological features of squamous differentiation and correlate with immunohistochemistry for p40. Level of p40 expression was correlated with outcome to evaluate whether p40 may be of prognostic value.

## Material and methods

The study was approved by a UK NHS Health Research Authority Research (HRA) Research Tissue Bank (CEPA Biobank, Newcastle upon Tyne Hospitals NHS Foundation Trust, Reference number: 17/NE0070). The Research Tissue Bank released link-anonymised patient tissue, surplus to diagnostic requirements, in accordance with the term of the ethical approval. The need for informed consent was waived by the Tissue Access and Governance Committee of the CEPA Biobank, Newcastle upon Tyne Hospitals NHS Foundation Trust, Reference number 17/NE0070. The p40 immunohistochemistry was performed according to validated protocols of an ISO15189:2012 accredited hospital laboratory.

### Study samples

117 consecutive, pancreatic EUS fine needle biopsy cases with a diagnosis of adenocarcinoma from 2015 were retrospectively identified from the laboratory system at the Department of Cellular Pathology at Newcastle upon Tyne Hospitals, UK. The following 10 cases were excluded: definite or possible diagnosis of metastatic adenocarcinoma, ampullary or common bile duct adenocarcinomas on subsequent resection histology, adenocarcinomas apparently arising in the context of intraductal papillary mucinous neoplasms (IPMNs) and adenocarcinomas with non-pancreatobiliary histological type. 16 matching resection specimens from FNB samples containing PDAC were also identified.

### Pathological assessment

Original archival diagnostic slides were assessed by a specialist pancreatic pathologist (BH) to confirm the diagnosis of pancreato-biliary phenotype adenocarcinoma, evaluate sufficiency of the material for immunohistochemistry for p40 and assess for morphological evidence of squamous differentiation. Diagnosis of adenocarcinoma was confirmed by morphological presence of glandular/acinar structures and, in rare cases, originally performed mucin stain (Alcian Blue Periodic Acid Schiff treated with Diastase as per local protocol). In each case tumour tissue was assessed for the three defining features of squamous differentiation: keratinisation, squamous pearls/eddies and intercellular bridges^17^. Further four cytomorphological features described and observed as being associated with squamous differentiation were also evaluated: presence of ovoid/elongated cell contours/shapes, sharply defined cell borders, a dense cytoplasm and central, squared/rectangular nuclei with homogenous dense chromatin and absent nucleoli^17^. Each of these morphological findings was assessed as absent or present in < 1/3, 1/3–2/3 and > 2/3 of tumour cells. In addition, focal and abundant presence or absence of necrosis and pyknotic/anucleate cells was noted. The 16 resection specimens from patients who had undergone an operation were reviewed and the diagnosis of pancreatic ductal adenocarcinoma confirmed. Two of the resections had been performed following neoadjuvant chemotherapy which led to significant tumour regression and altered morphology. Those were therefore excluded from the analysis.

### Immunohistochemistry

Excluding one further case with insufficient tissue remaining, immunohistochemistry for p40 using Ventana pre-diluted antibody P40 clone BC28 monoclonal mouse antibody (Ventana medical systems, Arizona, USA) was performed on 106 EUS samples as well as one representative block of the corresponding resection in 14 of the patients confirmed as primary pancreatic ductal adenocarcinoma. Slides were stained according to standardised protocol (Discovery ULTRA Staining Module, protocol 459) on Ventana Discovery Ultra IHC autostainer (Ventana medical systems, Arizona, USA) and included positive and negative controls.

The antigen retrieval was done on the Discovery Ultra IHC autostainer (Ventana medical systems, Arizona, USA) using CC1 (EDTA) for 64 min. Slides were incubated with the primary antibody for 32 min. The antibody was amplified using Ventana AMP multimer (Ventana medical systems, Arizona, USA), detected using Ventana ULTRAview Multimer (secondary) (Ventana medical systems, Arizona, USA) and DAB chromogen to visualise (Ventana medical systems, Arizona, USA).

All immunohistochemical stained sections were evaluated for p40 nuclear positivity in tumour cells by a specialist pancreatic pathologist (BH), independently of the previous morphological assessment. Nuclear staining intensity was assessed as follows: 0 = negative, 1 = weak, 2 = moderate and 3 = strong staining intensity. The number of positive nuclei was counted with only very large number of positive nuclei estimated +/− 50–100. Estimating the overall number of tumour cells in the sample semiquantatively, percentages of p40 positive nuclei were calculated. P40 H-score was calculated as the product of the percentage of p40 positive nuclei and the staining intensity (0–300). A p40 H-score of 0 indicated no presence of p40 expression, a score of e.g. 3 equated to 1% 3 + strongly stained nuclei or up to 3% weakly stained nuclei (see supplementary Fig. S1 online).

### Patient demographics and clinical data

The following clinical and demographic patient details were retrieved from hospital records of local and regional hospitals in the North East of England and recorded: age, gender, level of operability at presentation (operable, borderline, locally advanced or metastatic), surgical resection performed or not, type of chemotherapy treatment (none, adjuvant, palliative or neoadjuvant) and mortality.

### Statistical analysis

Evaluation of P40 H-score in different groups related to morphological, demographic and treatment features were examined using the Mann–Whitney test for comparisons between groups with two categories. The Kruskal–Wallis was used to compare between factors with three or more groups. P40 H-scores were classed into four statistically comparable groups to facilitate analysis of association with survival (see supplementary Fig. S1 online). Patient survival times were analysed using Kaplan–Meier methods. The logrank test was used to statistically compare the survival times between p40 H-score groups. A P-value of < 0.05 was considered to be statistically significant. Comparison of p40 H-score between EUS FNB samples and corresponding resections was performed using scatterplots and intra-class correlation method.

### Ethical approval

The tissues for this study were released by the CEPA Biobank, Newcastle upon Tyne Healthcare NHS Trust, UK, REC 17/NE0070.

### Consent for publication

All authors consent to the manuscript to be published in its current form. This is an original article. It has been previously presented as poster abstract at 51st Annual Meeting of the European Pancreatic Club, Bergen, Norway, 2019.

## Results

### Patient demographics and clinical data

53/106 (50%) patients were male. The mean age was 69.8 years (standard deviation 8.3 years; range = 45 to 89. 81 (76%) patients were inoperable at presentation and only 16 of 106 patients (15%) ultimately had a resection performed. 53 (50%) patients received palliative chemotherapy, 12 (11%) neoadjuvant and 14 (13%) adjuvant chemotherapy.

### P40 expression in PDAC FNBs

P40 expression was found in 64 (60%) of 106 PDAC FNBs. P40 H-score ranged from 1 to 240. P40 score was found to be highly skewed towards smaller values with few higher values and based upon defining statistically comparable groups p40 score was stratified into four groups: 0, 1–3, 4–30 and greater than 30 (see supplementary Fig. S1 online). P40 was found to be 0 in 42 (40%), 1–3 in 33 (31%), 4–30 in 17 (16%) and > 30 in 14 (13%) of cases.

### Morphological features and p40 H-score

**Figure 1:**
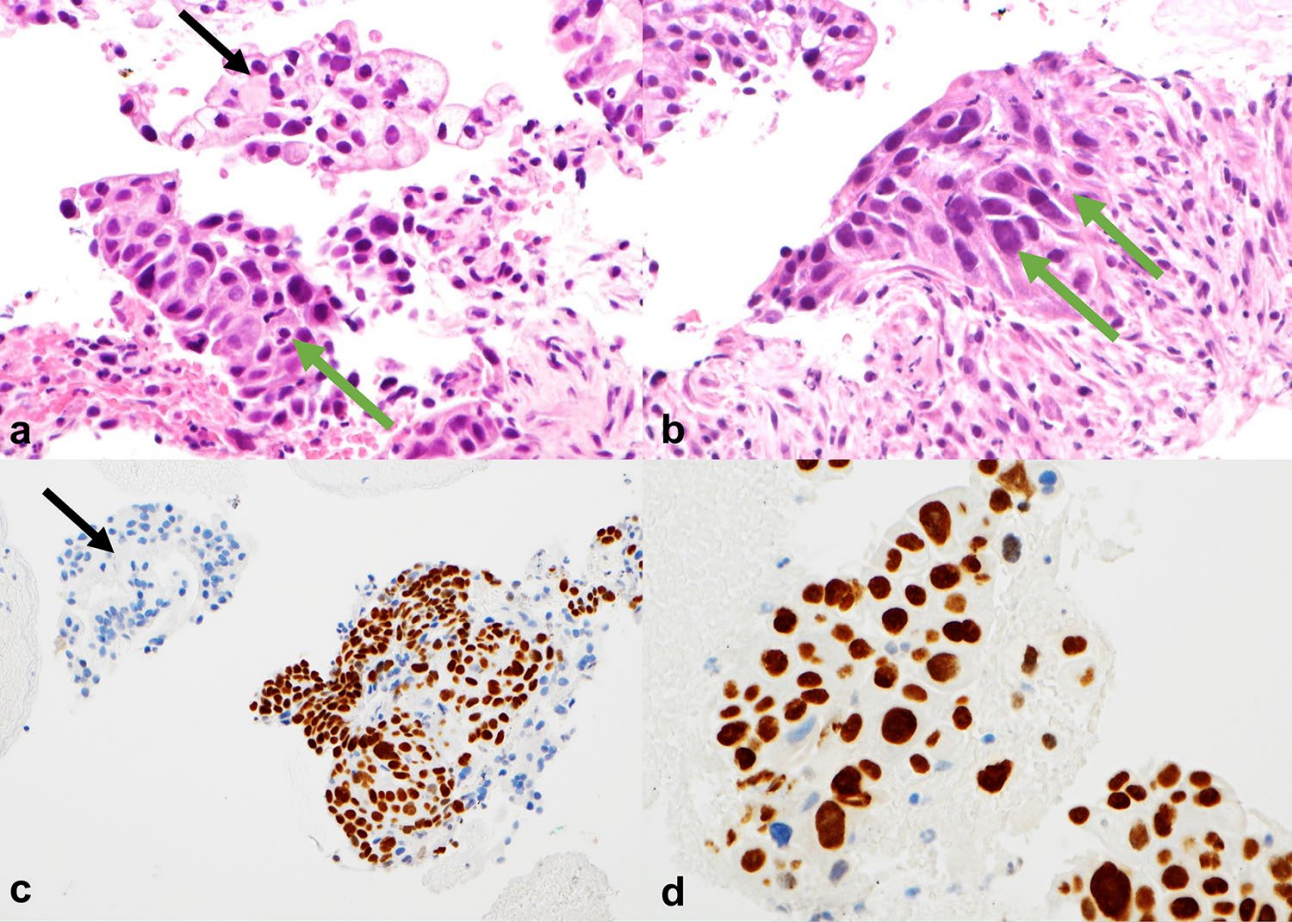
EUS FNB sample morphology (haematoxylin and eosin) and immunohistochemical p40 expression: (**a**) and (**b**) PDAC with intercellular bridges, elongated cells, sharply defined cell borders, dense cytoplasm and angular nuclei with homogenous chromatin (green arrows). Conventional atypical glandular epithelium (black arrows). (**c**) and (**d**) p40 nuclear expression.

Keratinisation was not observed and squamous eddies/pearls only in one out of 106 cases (0.9%). Intercellular bridges were seen in 12 cases (11.3%). Only few cases were assessed as having specific morphological features present in 1/3–2/3 and > 2/3 of tumour cells and therefore all specific morphological features were assessed as present or absent with the exception of necrosis. Presence of (1) intercellular bridges, (2) ovoid/elongated cell contours/shapes, (3) sharply defined cell borders and (4) central, squared/rectangular nuclei with homogenous dense chromatin without nucleoli were all significantly and strongly associated with p40 H-score (p < 0.001) as was (5) dense cytoplasm (p = 0.002). Pyknosis or level of necrosis did not show significant association with p40 H-score (Table 1). Morphological features and p40 nuclear expression are demonstrated in Fig. 1.

**Table 1:** Associations between morphological features and FNB p40 H-score.

Variable	Category	N	Staining intensity Mean ± SD	% positive cells Median [IQR]	P40 H-score Median [IQR]	P-value
Keratinisation	Absent	106	1.7 ± 1.4	0.5 [0, 2]	1 [0, 6]	–
	Present	0			–	
Pearls/Squamous eddies	Absent	105	1.6 ± 1.4	0.5 [0, 2]	1 [0, 6]	–
	Present	1	3.0 ± 0.0	0.5 [0.5, 0.5]	2 [2]	
Intercellular bridges	Absent	94	1.5 ± 1.4	0.5 [0, 1]	1 [0, 3]	** < 0.001**
	Present	12	2.6 ± 0.9	14 [6, 23]	38 [18, 66]	
Elongated cell shape	Absent	63	1.3 ± 1.4	0 [0, 1]	0 [0, 2]	** < 0.001**
	Present	43	2.1 ± 1.2	1 [0.5, 11]	3 [1, 33]	
Sharply defined cell border	Absent	65	1.3 ± 1.4	0 [0, 1]	0 [0, 2]	** < 0.001**
	Present	41	2.2 ± 1.2	1 [0.5, 12]	3 [1, 36]	
Dense cytoplasm	Absent	56	1.3 ± 1.4	0 [0, 1]	0 [0, 2]	**0.002**
	Present	50	2.0 ± 1.3	1 [0, 8]	2 [0, 24]	
Rectangular nuclei with homogenous chromatin	Absent	64	1.1 ± 1.4	0 [0, 1]	0 [0, 2]	** < 0.001**
	Present	42	2.4 ± 1.1	1 [0.5, 12]	3 [1, 36]	
Pyknosis/anucleosis	Absent	95	1.6 ± 1.4	0.5 [0, 2]	1 [0, 6]	0.11
	Present	11	2.4 ± 1.2	0.5 [0.5, 13]	2 [1, 39]	
Level of necrosis	Absent	7	2.1 ± 1.5	2 [0, 12]	6 [0, 36]	0.31
	Little	79	1.5 ± 1.4	0.5 [0, 2]	1 [0, 6]	
	Abundant	20	2.0 ± 1.4	0.5 [0, 2]	2 [0, 6]	

Bold indicates correspond to statistically significant P values of less than 0.05

### Comparison of EUS FNBs and corresponding resections

14 resected pancreatic ductal adenocarcinoma without prior neoadjuvant chemotherapy were included in the analysis for comparison between EUS FNB samples and corresponding resection specimens. None of the resections showed a specific subtype of pancreatic ductal adenocarcinoma, in particular none fulfilled criteria of adenosquamous carcinoma or showed convincing keratinisation. Because of the small numbers of resection specimens, evaluation of specific cytomorphological features as performed on the EUS FNB samples was not conducted as the number was too small for meaningful statistical analysis.

P40 expression was seen in 8 of 14 PDAC resections (57%) with p40 H-score of 1–3 in 6 cases (43%) and 4–30 in 2 cases (14%). When comparing P40 H-score of FNBs and corresponding resections, the scatter plot showed relatively good agreement for low H-scores but larger values showed less good agreement (see supplementary Fig. S2 online). The intra-class correlation value was 0.31 with 95% confidence interval from 0.00 to 0.71 suggesting fairly poor agreement between the H-score in FNB and resection specimens.

### Demographic and clinical features and p40 H-score

There was no significant association of p40 H-score with age, gender, chemotherapy or operability. A slight association with surgical resection was found but this did not reach statistical significance (p = 0.06) (Table 2).

**Table 2:** Associations between demographic/treatment factors and FNB p40 H-score.

Variable	Category	N	P40 H-score Median [IQR]	P-value
Age	≤ 65	26	1 [0, 6]	0.39
	66–75	51	2 [0, 15]	
	> 75	29	1 [0, 2]	
Gender	Female	53	1 [0, 2]	0.25
	Male	53	2 [0, 6]	
Operability	Borderline	22	2 [0, 15]	0.35
	Locally advanced	38	1 [0, 2]	
	Metastatic	21	2 [0, 3]	
	Operable	25	2 [0, 9]	
Operability* (binary)	Inoperable*	81	1 [0, 6]	0.37
	Operable	25	2 [0, 9]	
Resection**	No**	90	1 [0, 6]	0.06
	Yes	16	3 [1, 12]	
Chemotherapy	None	27	1 [0, 21]	0.30
	Palliative	53	1 [1, 6]	
	Adjuvant	14	3 [2, 12]	
	Neo-adjuvant	12	1 [0, 8]	

*Inoperable combines patients with borderline, locally advanced and metastatic PDAC.

**No resection includes all inoperable patients and patients who did not undergo an operation for reasons unrelated to PDAC.

### Patient survival by H-score

**Figure 2:**
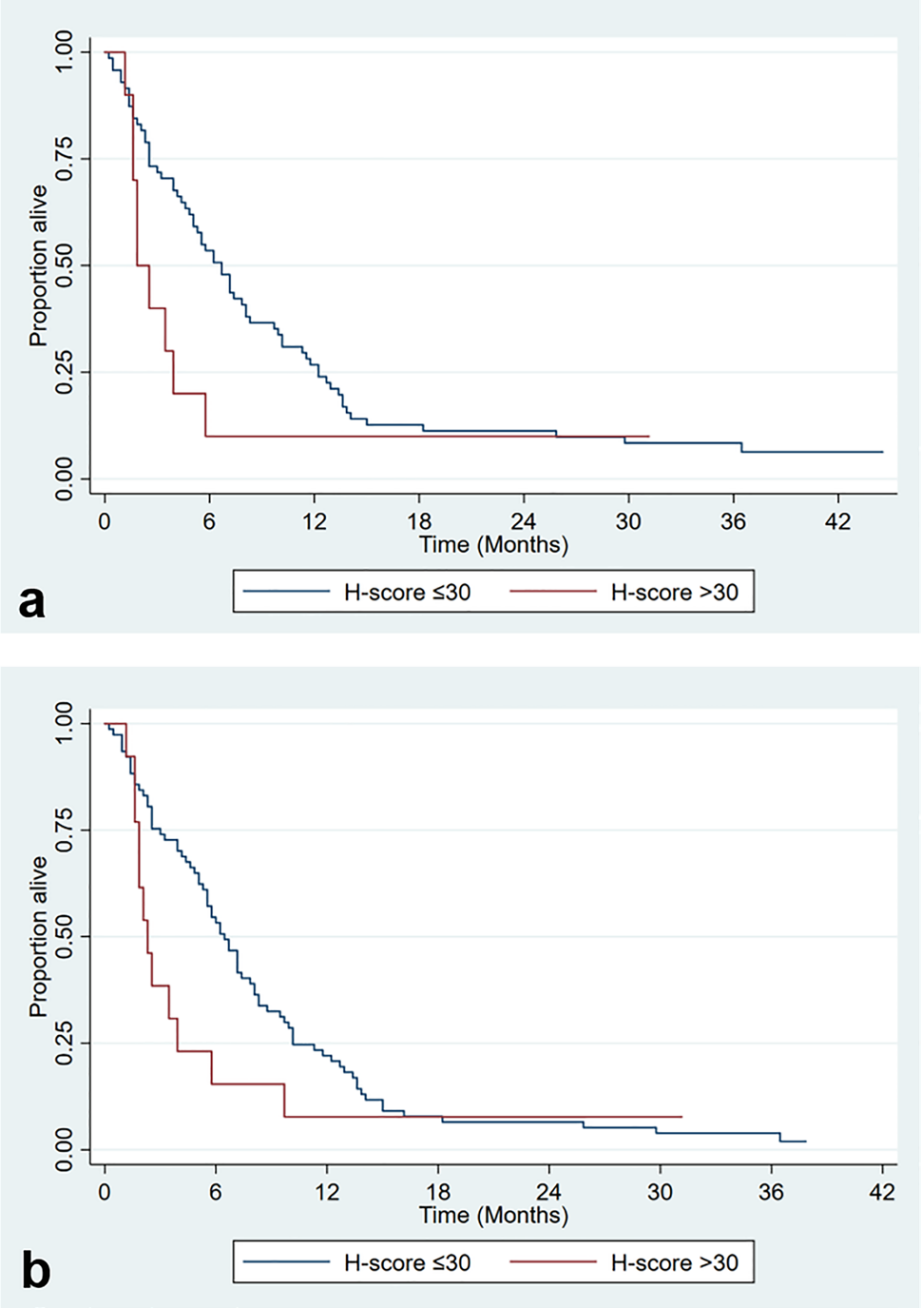
Kaplan–Meier plot of survival times by FNB p40 H-score in (**a**) inoperable patients, (**b**) non-resected patients.

Comparing four patient groups with p40 H-score 0, 1–3, 4–30 and > 30 or two patient groups with p40 H-score 0–30 and > 30; no significant difference in survival was found. Considering only inoperable patients (N = 81) a shorter survival was observed in patients with p40 H-score > 30 compared to those with p40 H-score 0–30 (1.8 months versus 6.7 months respectively). A similar result was observed when evaluating outcome for non-resected patients (90), including those who were radiologically operable but had other contraindications for surgery: 2.3 months overall survival with p40 H-score > 30 versus 6.5 months with p40 H-score 0–30). This did not reach statistical significance (p = 0.08) (Table 3, Fig. 2a and b).

**Table 3:** Patient survival by FNB p40 H-score.

Patient group	p40 H-score	N	Survival (months) Median (95% CI)	P-value
All patients (N = 106)	0	42	6.5 (3.2, 8.8)	0.45
	1–3	33	6.7 (5.5, 9.5)	
	4–30	17	9.9 (5.1, 13.4)	
	> 30	14	2.3 (1.6, 5.8)	
All patients (N = 106)	≤ 30	92	7.2 (5.8, 8.8)	0.24
	> 30	14	2.3 (1.6, 5.8)	
Inoperable	≤ 30	66	6.7 (4.8, 8.1)	0.08
Patients (N = 75)	> 30	9	1.8 (1.2, 3.9)	
Patients not	≤ 30	77	6.5 (5.3, 7.8)	0.08
Resected (N = 90)	> 30	13	2.3 (1.6, 3.9)	
Patients not resected & no chemotherapy (N = 27)	≤ 30	22	3.9 (1.6, 6.5)	0.24
	> 30	5	1.8 (1.6, +)

When considering patients who had not undergone an operation and had not received chemotherapy (N = 27) a shorter median survival was also seen with p40 H-score > 30 (2.3 months, N = 5) compared with p40 H-score 0–30 (3.9 months, N = 22) but this was not statistically significant (p = 0.24). (Table 3).

## Discussion

In a cohort of over 100 consecutive PDAC FNB samples, we found p40 to be a reliable immunohistochemical marker of squamous differentiation^18^ to be expressed in over half of the samples, with 13% showing expression in at least 10% of tumour cells. Immunohistochemical evidence of squamous differentiation was closely associated with specific cytomorphological features found in squamous carcinomas^17^ despite absence of keratinisation and squamous pearls. P40 H-score of greater 30 was associated with a shorter survival in patients without operation, although this did not quite reach statistical significance and did not consider type, dose and duration of chemotherapy given. P40 H-score greater 30 in patients who did not receive chemotherapy was also associated with shorter survival but this was not statistically significant, only affected a small group of patients and did not consider clinical stage of the disease. The corresponding resections in 14 patients without neoadjuvant chemotherapy showed p40 expression in over half of the samples, very similar to the FNBs but with poor agreement in paired samples with higher p40 H-scores. To the best of our knowledge systematic immunohistochemical evaluation of p40 on PDAC FNBs has not been performed before. FNBs provide a unique insight into the histomorphology of non-resected PDACs and represent well preserved tissue samples for examination of potential biomarkers. This would allow prognostication in the nearly 80% of patients with signs and symptoms of PDAC who are not undergoing an operation, and could help to guide their treatment, particularly in the era of precision medicine.

Squamous differentiation in pancreatic carcinoma has been observed for more than a century^19^. It finds recognition in adenosquamous carcinoma of pancreas (ASCP), a subtype of PDAC, arbitrarily defined as containing more than 30% squamous component based on routine histological assessment^20^. ASCP is rare accounting for approximately 3–4% of PDACs^21,22^, has been shown to be associated with a poorer prognosis^13,22,23^ but is not treated differentially. As the diagnosis rests on the subjective morphological identification of squamous differentiation by the histopathologist and depends on the amount of tumour tissue examined, it will be heavily influenced by varying practices of specimen assessment. In addition, the definition cannot be applied reliably to biopsies as they may not be representative of the whole tumour. Some studies suggest that the poorer prognosis of ASCP may be independent of the amount of squamous differentiation present, indicating that any squamous differentiation may be of prognostic and clinical importance^22,23^. Ito et al. found a squamous component greater than 60% was associated with worse survival^24^. Immunohistochemical markers for squamous differentiation are not routinely used for the diagnosis of ASCP although deltaNp63 (p40) has been shown to be a reliable and specific marker of squamous differentiation in neoplastic and non-neoplastic pancreatic tissue^16^.

The relatively recently identified basal-like/quasi-mesenchymal/squamous epithelial molecular subtype of PDAC with poorer prognosis may account for as much as 25% of PDACs. This raises the possibility that squamous differentiation in PDAC may be of greater clinical importance, but only a limited association of this molecular subtype with ASCP has been found^8^. Conversely, molecular analyses of histologically confirmed ASCP have found major genomic similarities to glandular PDAC but with a greater enrichment in p53 mutations and 3p loss^25^. There are emerging morphological classifications based on gland percentage^26^ and immunohistochemical markers such as GLI1^12,27^ for basal-like and GATA6^12,28^ for classical that may predict molecular subtypes of PDAC. However, these have been mainly applied to resected specimens and their use in diagnostic samples may be limited. In addition, stromal component and microenvironment in PDAC may also impact on outcome^12^. Furthermore, recent studies suggest that basal-like and classical molecular subtypes coexist in the same tumour with epigenetically driven TP63 based reprogramming causing squamous transdifferentiation associated with tumour aggressiveness, including increased invasiveness and tumour size^29–31^. The basal-like molecular subtype may therefore represent clonal transdifferentiation to a more aggressive PDAC phenotype rather than a unique, easily reproducible molecular and histomorphological subtype. There is evidence that the molecular classification of PDAC is along a continuous gradient with pure classical and pure basal-like subtypes being at either end of a continuous spectrum^32^. This molecular heterogeneity of PDAC appears to be driven by epigenetic phenomena^30,32,33^ with plasticity potentially allowing tumour cells to transition from one molecular subtype to another^33^. Basal-like molecular signatures have been shown to be associated with squamoid morphological features/squamous differentiation^30^. Specifically targeting activation of TP63ΔN (p40) network may prevent/revert transdifferentiation to basal-like type and open therapeutic avenues^29,33^. Selective expression of PD-L1 in the squamous component of ASCP in two studies^34,35^ may also potentially suggest squamous differentiation as therapeutic target for immune checkpoint inhibitors.

Our study shows that immunohistochemistry for squamous differentiation can be reliably performed on EUS FNB samples of PDAC and may have the potential to provide prognostic information and inform treatment decisions, particularly in patients who are not undergoing resection. Our results add to the growing evidence that squamous differentiation may play an important role in PDAC. They indicate that immunohistochemical evidence of squamous differentiation correlates with specific cytomorphological features in diagnostic biopsies allowing the practicing pathologist to recognise a characteristic morphological picture associated with more aggressive phenotype PDAC, even in diagnostic biopsy material. The use of a cohort of consecutive, high quality PDAC FNB samples from treatment naïve patients and the detailed evaluation by an experienced specialist pancreatic pathologist strengthen our results. Significant limitations of our study include the single centre, retrospective nature of our study, the overall small sample size which limits statistical power and prohibits multivariate analysis, the sample evaluation by a single pathologist and the lack of corresponding molecular analyses. Additionally, comparison of FNB samples and corresponding resections was limited to a small number showing overall poor agreement which requires further systematic and more detailed exploration.

In conclusion, immunohistochemical evidence of squamous differentiation is found in a significant proportion of FNB samples with PDAC and corresponding resections. P40 expression in FNBs with PDAC is associated with specific cytomorphological features and a shorter survival in inoperable/non-operated patients. Squamous differentiation may also represent a treatment target making its assessment in diagnostic biopsies from inoperable patients highly valuable. Larger future studies are required to further validate and clearly define the role of squamous differentiation in diagnostic biopsies for histological subtyping, prognosis and treatment of PDAC.

## Supplementary Information

Supplementary Information.

## Data Availability

Datasets analysed during the current study are available from the corresponding author in reasonable request.

## References

[1] Ilic M, Ilic I (2016). Epidemiology of pancreatic cancer. World J. Gastroenterol..

[2] Cancer Research UK. *Pancreatic cancer incidence statistics.* https://www.cancerresearchuk.org/health-professional/cancer-statistics/statistics-by-cancer-type/pancreatic-cancer. (2017, accessed June 2020).

[3] Rahib L (2014). Projecting cancer incidence and deaths to 2030: The unexpected burden of thyroid, liver, and pancreas cancers in the United States. Cancer Res..

[4] Karanikas M (2016). Pancreatic Cancer from Molecular Pathways to Treatment Opinion. J. Cancer.

[5] Karamitopoulou E, Gloor B (2019). Clinical scenarios emerging from combined immunophenotypic, molecular and morphologic analysis of pancreatic cancer: The good, the bad and the ugly scenario. Cancers (Basel).

[6] Collisson EA (2011). Subtypes of pancreatic ductal adenocarcinoma and their differing responses to therapy. Nat. Med..

[7] Moffitt RA (2015). Virtual microdissection identifies distinct tumor- and stroma-specific subtypes of pancreatic ductal adenocarcinoma. Nat. Genet..

[8] Bailey P (2016). Genomic analyses identify molecular subtypes of pancreatic cancer. Nature.

[9] Cancer Genome Atlas Research Network (2017). Integrated Genomic Characterization of Pancreatic Ductal Adenocarcinoma. Cancer Cell.

[10] Aguirre AJ (2018). Refining Classification of Pancreatic Cancer Subtypes to Improve Clinical Care. Gastroenterology.

[11] Maurer C (2019). Experimental microdissection enables functional harmonisation of pancreatic cancer subtypes. Gut.

[12] Puleo F (2018). Stratification of Pancreatic Ductal Adenocarcinomas Based on Tumor and Microenvironment Features. Gastroenterology.

[13] Boyd CA, Benarroch-Gampel J, Sheffield KM, Cooksley CD, Riall TS (2012). 415 Patients with Adenosquamous Carcinoma of the Pancreas: A Population-Based Analysis of Prognosis and Survival. J. Surg. Res..

[14] Eltoum IA, Alston EA, Robertson J (2012). Trends in pancreatic pathology practice before and after implementation of endoscopic ultrasound-guided fine-needle aspiration: An example of disruptive innovation effect?. Arch. Pathol. Lab. Med..

[15] Oppong KW (2020). Fork-tip needle biopsy versus fine-needle aspiration in endoscopic ultrasound-guided sampling of solid pancreatic masses: A randomized crossover study. Endoscopy.

[16] Basturk O (2005). DeltaNp63 expression in pancreas and pancreatic neoplasia. Mod. Pathol..

[17] Travis WD (2013). Diagnosis of Lung Cancer in Small Biopsies and Cytology – Implications of the 2011 International Association for the Study of Lung Cancer/American Thoracic Society/European Respiratory Society Classification. Arch. Pathol. Lab. Med..

[18] Nonaka D (2012). A study of ΔNp63 expression in lung non-small cell carcinomas. Am. J. Surg. Pathol..

[19] Borazanci E (2015). Adenosquamous Carcinoma of the Pancreas: Molecular Characterization of 23 Patients Along With a Literature Review. World. J. Gastrointest. Oncol..

[20] Hruban, R. H. *et al*. Pancreatic ductal adenocarcinoma. In *WHO Classification of Tumours: Digestive System Tumours, 5th edn*. (ed. WHO Classification of Tumours Editorial Board) 327–328 (International Agency for Research on Cancer, Lyon, 2019)

[21] Morohoshi T, Held G, Klöppel G (1983). Exocrine pancreatic tumours and their histological classification. A study based on 167 autopsy and 97 surgical cases. Histopathology.

[22] Voong KR (2010). Resected pancreatic adenosquamous carcinoma: Clinicopathologic review and evaluation of adjuvant chemotherapy and radiation in 38 patients. Hum. Pathol..

[23] Kardon DE, Thompson LD, Przygodzki RM, Heffess CS (2001). Adenosquamous carcinoma of the pancreas: A clinicopathologic series of 25 cases. Mod. Pathol..

[24] Ito T (2019). Long-term outcomes after an aggressive resection of adenosquamous carcinoma of the pancreas. Surg. Today.

[25] Fang Y (2017). Genomic signatures of pancreatic adenosquamous carcinoma (PASC). J. Pathol..

[26] Kalimuthu SN (2020). Morphological classification of pancreatic ductal adenocarcinoma that predicts molecular subtypes and correlates with clinical outcome. Gut.

[27] Muckenhuber A (2018). Pancreatic ductal adenocarcinoma subtyping using the biomarkers hepatocyte nuclear factor-1A and cytokeratin-81 correlates with outcome and treatment response. Clin. Cancer Res..

[28] Aung KL (2018). Genomics-driven precision medicine for advanced pancreatic cancer: Early results from the COMPASS trial. Clin. Cancer Res..

[29] Somerville T (2018). TP63-mediated enhancer reprogramming drives the squamous subtype of pancreatic ductal adenocarcinoma. Cell Rep..

[30] Hayashi, A. *et al*. The genetic basis of transcriptional heterogeneity of basal-like features in pancreatic ductal adenocarcinoma. *bioRxiv* 548354 (2019).

[31] Juiz, N. *et al.* Basal-like and Classical cells coexistence in pancreatic cancer revealed by single cell analysis on biopsy-derived pancreatic cancer organoids from the classical subtype. *FASEB J.* (2020) 10.1096/fj.202000363RR 32686876

[32] Nicolle R (2020). Establishment of a pancreatic adenocarcinoma molecular gradient (PAMG) that predicts the clinical outcome of pancreatic cancer. EBioMedicine.

[33] Andricovich J (2018). Loss of KDM6A activates super-enhancers to induce gender-specific squamous-like pancreatic cancer and confers sensitivity to BET inhibitors. Cancer Cell.

[34] Tanigawa M (2018). PD-L1 expression in pancreatic adenosquamous carcinoma: PD-L1 expression is limited to the squamous component. Pathol. Res. Pract..

[35] Silvestris N (2018). Immunological mutational signature in adenosquamous cancer of pancreas: An exploratory study of potentially therapeutic targets. Expert Opin. Ther. Targets.

