# Supplementary material for: Morphological and p40 immunohistochemical analysis of squamous differentiation in endoscopic ultrasound guided fine needle biopsies of pancreatic ductal adenocarcinoma
Source: Sci Rep. 2021 Oct 28;11:21290. doi: 10.1038/s41598-021-00652-5 (PMC8553828; doi:10.1038/s41598-021-00652-5)
Supplement: Supplementary file 1 — Supplementary Information. [file 41598_2021_652_MOESM1_ESM.pdf]

Morphological and p40 immunohistochemical analysis of squamous differentiation in endoscopic ultrasound guided fine needle biopsies of pancreatic ductal adenocarcinoma

Beate Haugk*[1], David Horton[2], Kofi Oppong[2], John Leeds[2], Antony Darne[1], Philip Sloan[1], Thomas Ness[1], Claire Jones[1], Paul Bassett[3], Manu Nayar[2]

1. Department of Cellular Pathology, Royal Victoria Infirmary, Newcastle upon Tyne Hospitals NHS Foundation Trust, Newcastle upon Tyne, United Kingdom.
2. HPB Unit, Freeman Hospital, Newcastle upon Tyne Hospitals NHS Foundation Trust, Newcastle upon Tyne, United Kingdom.
3. Statsconsultancy Ltd, Amersham, Bucks, United Kingdom.

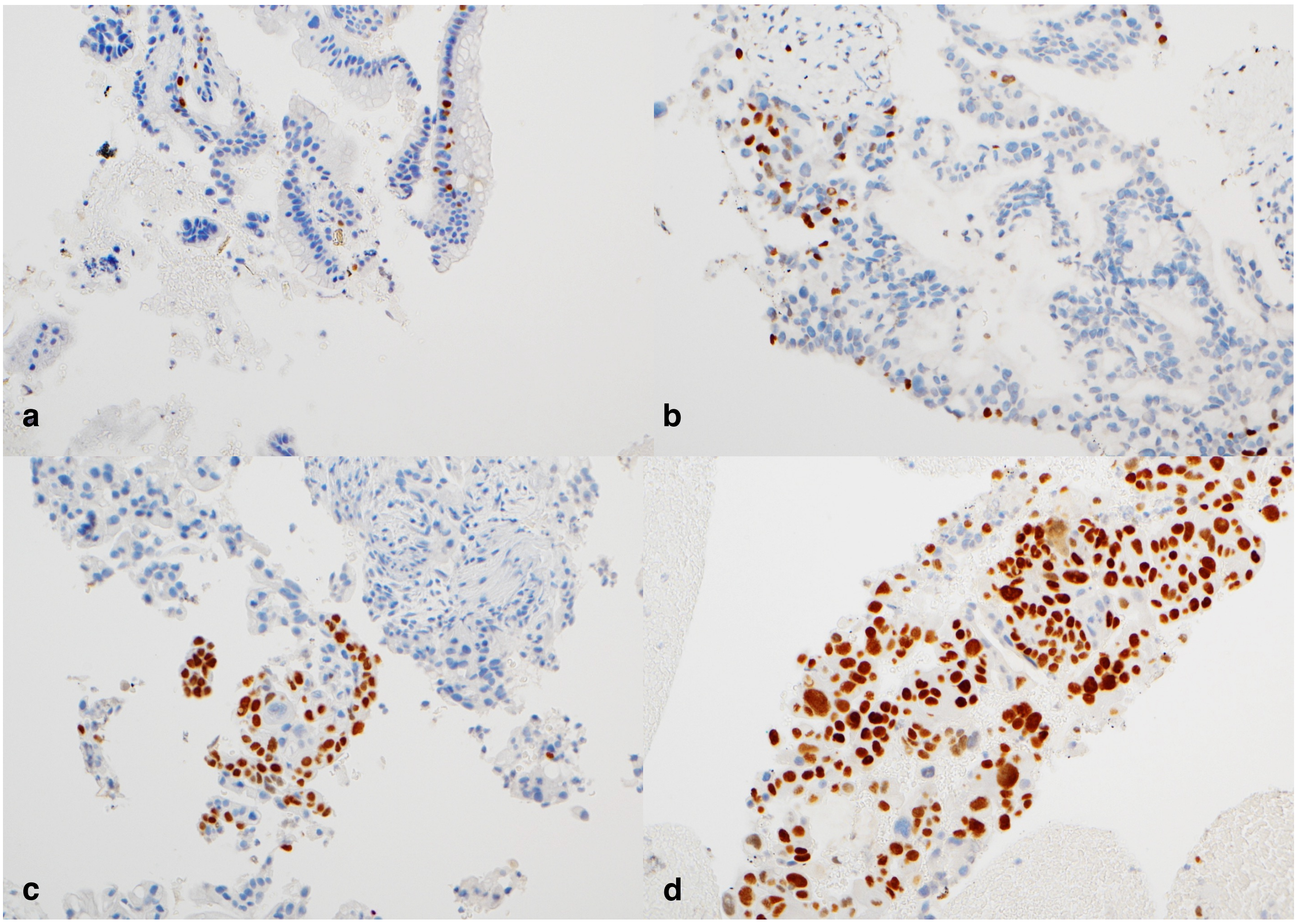

SUPPLEMENTARY FIGURE S1: EUS FNBs p40 H-scores: a: 3 (1% nuclei, staining intensity 3), b: 18 (6% nuclei, staining intensity 3), c: 75 (25% nuclei, staining intensity 3), d: 240 (80% nuclei, staining intensity 3)

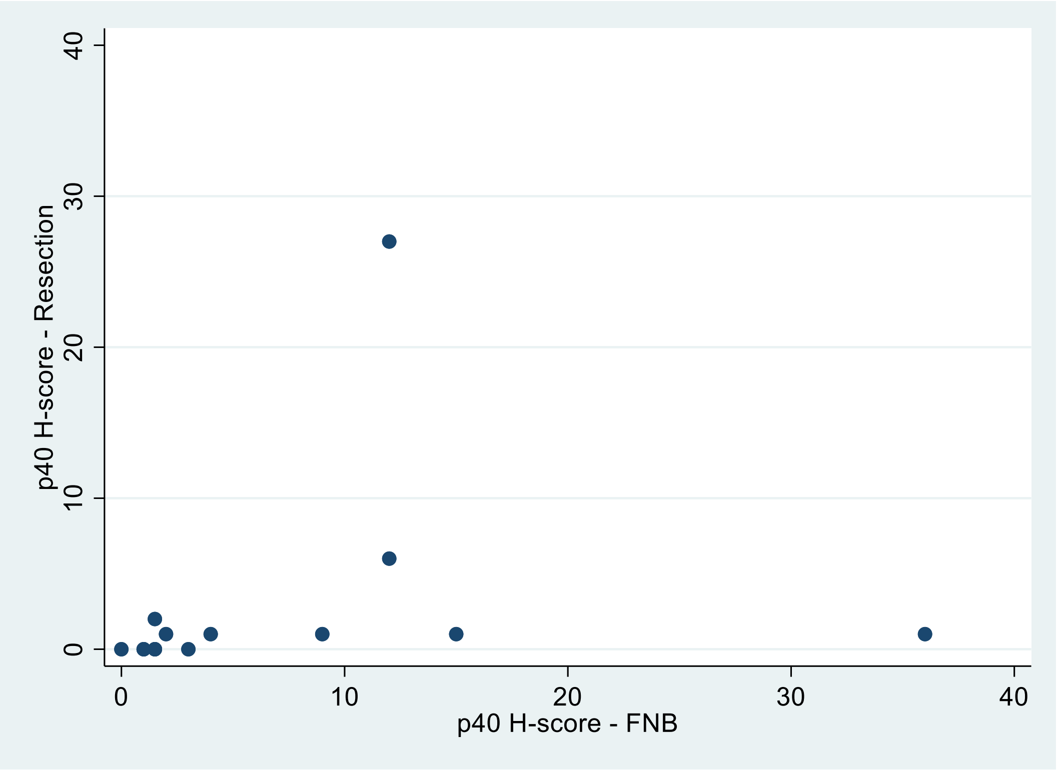

SUPPLEMENTARY FIGURE S2: Scatterplot of p-40 H-scores of 14 paired FNBs and resections
